# Spatial analysis of the association of alcohol outlets and alcohol-related pedestrian/bicyclist injuries in New York City

**DOI:** 10.1186/s40621-016-0076-5

**Published:** 2016-04-04

**Authors:** Charles DiMaggio, Stephen Mooney, Spiros Frangos, Stephen Wall

**Affiliations:** 1 Department of Surgery, Division of Trauma and Acute Care Surgery, New York University School of Medicine, 550 First Avenue, New York, NY 10016 USA; 2 Mailman School of Public Health, Epidemiology Department, Columbia University, 720 West 168 St, New York, NY 10032 USA; 3 Ronald Pearlman Department of Emergency Medicine, New York University School of Medicine, 550 First Avenue, New York, NY 10016 USA

## Abstract

**Background:**

Pedestrian and bicyclist injury is an important public health issue. The retail environment, particularly the presence of alcohol outlets, may contribute the the risk of pedestrian or bicyclist injury, but this association is poorly understood.

**Methods:**

This study quantifies the spatial risk of alcohol-related pedestrian injury in New York City at the census tract level over a recent 10-year period using a Bayesian hierarchical spatial regression model with Integrated Nested Laplace approximations. The analysis measures local risk, and estimates the association between the presence of alcohol outlets in a census tract and alcohol-involved pedestrian/bicyclist injury after controlling for social, economic and traffic-related variables.

**Results:**

Holding all other covariates to zero and adjusting for both random and spatial variation, the presence of at least one alcohol outlet in a census tract increased the risk of a pedestrian or bicyclist being struck by a car by 47 % (IDR = 1.47, 95 % Credible Interval (CrI) 1.13, 1.91).

**Conclusions:**

The presence of one or more alcohol outlets in a census tract in an urban environment increases the risk of bicyclist/pedestrian injury in important and meaningful ways. Identifying areas of increased risk due to alcohol allows the targeting of interventions to prevent and control alcohol-related pedestrian and bicyclist injuries.

## Background

After many years of declines, pedestrian and bicyclist injuries have been increasing across the United States (Patek and Thoma 2013; NHTSA 2011). Active transportation, including walking, accounts for 2.8 % of all commutes and 8.6 % of all trips in the US, but pedestrians represented 11.3 % (4,645) of the 41,059 total US traffic fatalities in 2007 (FHWA 2010; NHTSA 2007).

Pedestrian injury and fatality is of particular importance in dense urban environments. In 2007 73 % of pedestrian crashes in the US occurred in urban areas (NHTSA 2007). In New York City, pedestrian deaths have outnumbered motor-vehicle occupant deaths since 1910, and between 1997 and 2006, New York City accounted for the largest proportion of pedestrian fatalities in the United States (Chang 2014). Recently, the office of the mayor has instituted a “Vision Zero” traffic plan to address the “epidemic of traffic fatalities and injuries” in New York City (NYC 2015). The initiative is based on pioneering efforts in Sweden to bring traffic fatalities down to zero (Sweden 2015), and focuses in large part on pedestrians and bicyclists. The New York City plan involves educational, enforcement, and engineering interventions, as well as research, surveillance and data analysis “to help target traffic safety interventions” and evaluate the effectiveness of the program (NYC 2015).

Retail environments, particularly alcohol outlets, may contribute to the risk pedestrians face from traffic (Treno et al. 2007; LaScala et al. 2001; Campbell et al. 2009; Kuhlmann et al. 2009). In the United States, nearly half of car crashes in which a pedestrian is killed involve an intoxicated pedestrian or driver (Chang 2008). In New York City, 15 % of pedestrians and 10 % of bicyclists injured by motor vehicles had used alcohol as measured by emergency deparment blood alcohol testing (Dultz et al. 2013). Despite these individual-level associations, studies have found that proximity to bars is unrelated to alcohol consumption (Bernstein et al. 2007) and negatively associated with drinking and driving (Gruenewald et al. 2002). In this latter study, while bar density was not associated with drinking and driving, restaurant density was. A follow up study by the same group using similar methods later found bar densities to be positively associated with drinking and driving (Ponicki et al. 2013). Inconsistency in the observed relationship between alcohol outlets and both proximal and distal consequences may derive partially from differences in modeling proximity to alcohol outlets or the pedestrian population at risk. Bayesian hierarchical spatial regression models offer a natural modeling strategy to explore spatio-temporal differences in risk, and have been explored extensively to relate presence of alcohol outlets to prevalence of violent crime (Toomey et al. 2012; Yu 2008; Sparks 2011). To our knowledge, they have not previously been applied to investigate the relationship between alcohol outlets and pedestrian/bicyclist injury risk.

This analysis adds to and is part of a series of recent public health analyses focusing on built environment and its role in pedestrian injury in the context of Vison Zero initiatiatives in New York City. Among these studies are reports that “traditional engineering measures, in particular, signal-related ones, remain effective, when installed at appropriate locations” (Chen et al. 2013), the utility of remote imagery to characterise pedestrian injury risk (Mooney et al. 2016), and a series of articles documenting the safety benefits, and cost effectivness of Safe Routes to School environmental interventions in preventing pediatric pedestrian injury (DiMaggio and Li 2013; Muennig et al. 2014, DiMaggio et al. 2014).

This study quantifies the spatiotemporal risk of alcohol-related pedestrian injury in New York City at the census tract level over a recent 10-year period using a Bayesian hierarchical spatial regression model (Besag et al. 1991) with Integrated Nested Laplace approximations (Blangiardo et al. 2013; Rue et al. 2013). The study measures local risk, and estimates the association between the presence of alcohol outlets in a census tract and pedestrian/bicyclist injury after controlling for social, economic and traffic-related variables. The primary goals of the study are to quantify the role of alcohol outlets, which includes grocery, catering, and eating establishments in addition to bars, with pedestrian/bicyclist injury in a dense urban environment, and to establish an approach to evaluations of the built environment at the local level for alcohol-related pedestrian injury. Secondary goals are to quantify and interpret the association of ecologic-level variables that may contribute to pedestrian and bicyclist injury risk at the community level.

## Methods

Crash data for motor vehicle injuries to pedestrians and bicyclists were obtained from the New York City Department of Transportation. These were based on police investigations for all motor-vehicle crashes in New York City for the years 2001 to 2010 involving personal injury or property damage in excess of $1,000. Records were restricted to those with for which alcohol was listed as a primary contributing factor. These records do not indicate whether it was the driver or the pedestrian or bicyclist that was impaired by alcohol. Crash latitude and longitude coordinates were assigned to census tracts using the R maptools package (Lewin-Koh and Bivand 2012). Census tract populations were based on United States 2000 and 2010 decennial census enumerations (US Census Bureau 2010). Census tracts were restricted to 1,929 census tracts that were present in both 2000 and 2010, creating a closed cohort of census tracts. Population estimates were linearly interpolated over inter-census years. Census tract housing and economic data were based on 2010 US Census estimates. Alcohol outlet data were derived from street addresses for all currently active liquor licenses in New York City (NYSLA 2015a). There are 21 classes of liquor licenses covering such enteties as delicatessens, grocery stores, catering and eating establishments as well as bars (NYSLA 2015b). Outlet addresses were geocoded and each outlet was assigned to a census tract (Texas A and M University 2015). These data were merged with the pedestrian/bicyclist crash data files based on census tract.

A social fragmentation index (Congdon 2013; Pabayo et al. 2014) was created using 4 variables extracted from 2010 US census variables: the proportion of total housing units in a census tract that were not owner occupied, the proportion of vacant housing units, the proportion of individuals living alone and the proportion of housing units into which an occupant recently moved. A “recent” move in the Census data was defined as anytime after the prior census enumeration, i.e. 2000. The four component social fragmentation index variables were standardized and summed with equal weights, resulting in a normally distributed index with mean zero and 95 % quantiles between −2.5 and 2.2.

An economic measure was based on the census tract median household income for the past 12 months (in units of 10,000 2012 inflation-adjusted dollars). Census tract traffic-related variables were obtained from the New York City Department of Health and Mental Hygeine, and consisted of traffic density (vehicle kilometers traveled per day per square kilometer) in standardized units, and average speed in increments of 10 miles per hour (Ross et al. 2013).

A census tract map file for New York City was obtained from the US Census and was matched to aggregated census tract counts of pedestrian/bicyclist injuries and presence or absence of alcohol outlets. Twenty-one census tracts with zero population representing large parks, beaches, cemeteries and train yards were excluded from analysis. Three census tracts representing highly-trafficked tourist areas with small underlying populations (Grand Central Station, Penn Station, and Times Square) were adjusted based on hotel capacity, adding 16,000 transient residents to each area (Karmin 2013). A contiguous neighbor adjacency matrix graph was created for use with the conditional autoregression term in the model using the poly2nb function in the spdep R package (Bivand 2013).

Counts of pedestrian and bicyclist injuries in New York City census tracts were spatially modeled (Lawson et al. 2000; Lawson 2013; Banerjee et al. 2004) as:

- $$ {y}_i \sim Pois\left({\lambda}_i = {e}_i{\theta}_i\right) $$
- $$ log\left({\theta}_i\right) = \beta {x}_i + {\upsilon}_i + {\eta}_i $$
- $$ \upsilon \sim nl\left( 0,{\tau}_{\upsilon}\right) $$
- $$ \eta \sim nl\left({\eta}_{\delta },\ {\tau}_{\eta }/{\eta}_{\delta}\right) $$

where,

1.  the *y*_*i*_ counts in area *i*, are independently identically Poisson distributed and have an expectation in area *i* of *e*_*i*_, the expected count, times *θ*_*i*_, the risk for area *i*.
2.  a logarithmic transformation (*log(θ*_*i*_*)*) allows a linear, additive model of regression terms (*βx*_*i*_), along with
3.  a spatially *unstructured* random effects component (*υ*_*i*_) that is i.i.d normally distributed with mean zero (*~ nl(0,τ*_*υ*_*)*), and
4.  a conditional autoregressive spatially structured component $\left(\upeta \sim nl\left({\overline{\upeta}}_{\updelta},{\uptau}_{\upeta}/{n}_{\updelta}\right)\right)$ in which a “neighborhood” consisting of spatially adjacent shapes is characterized by the normally distributed mean of the spatially structured random effect terms for the spatial shapes that make up the neighborhood $\left({\overline{\upeta}}_{\updelta}\right)$, and the standard deviation of that mean divided by the number of spatial shapes in the neighborhood (τ_η_/*n*_δ_). This spatially structured conditional autoregression component is also sometimes described as a Gaussian process λ ∼ *NI*(*W*, τ_λ_) where W represents the matrix of neighbors that defines the neighborhood structure, and the conditional distribution of each λ_*i*_, given all the other λ_*i*_ is normal with μ = the average λ of its neighbors and a precision (τ_λ_).

The baseline convolution model that consisted solely of an intercept term with unstructured and spatially structured random effect terms was extended to include covariates for the presence or absence of alcohol outlets in a census tract, social fragmentation, median household income, traffic density and average speed.

The final linear model consisted of an intercept (β_0_); and indicator variable for the presence or absence of alcohol outlets in a census tract (β_1_), a vector of census-level explanatory variables (β*x*_*i*_) for social fragmentation (Congdon index), economics (in $10,000 increments of median household income), average speed (in 10-mile per hour increments), and traffic density (in standardized units); a spatially unstructured random effect term (*υ*_*i*_); and a spatially structured conditional autoregression term (η_*i*_). The log of the yearly census-tract population was included as an offset variable.

In this model, the intercept is interpreted as the average city-wide per-population risk on the log scale adjusted for the presence of alcohol outlets, covariates, random effects and spatial terms. The exponentiated coefficient for the alcohol outlet term is interpreted as an incidence density ratio for the association of pedestrian/bicyclist injuries for census tracts with alcohol outlets vs. those without, controlling for the additional census-tract level explanatory covariates. The spatially unstructured random effect term captures normally-distributed or Gaussian random variation around the mean or intercept. The spatially-structured conditional autoregression term accounts for local geographic influence.

Spatial risk, controlling for or holding the covariates constant, was calculated as ζ_i_ = *υ*_*i*_ + η_*i*_, and is interpreted as the residual spatial risk for each area (compared to all of New York City) after presence of alcohol outlets, social fragmentation, economics, average speed, and traffic density are taken into account. The probability of spatial risk greater than 3 (Pr *e*_i_*ξ* >3) was calculated. These so-called exceedance probabilities (Clayton and Bernardinelli 1992) are the posterior probabilities for an area’s spatial risk estimate exceeding some pre-set value. This can be extended (Richardson et al. 2004) to decision rules “for classifying whether (an area) has an increased risk based on how much of the posterior distribution of the relative risk parameter … exceeds a reference threshold” (Best et al. 2005). They are calculated as the proportion of simulations for which the linear combination of effects (ζ) exceeds the target value. Lastly, the proportion of spatially explained variance was calculated as the proportion of total spatial heterogeneity accounted for by the spatially structured conditional autoregression variance.

Spatial modeling was conducted using integrated nested Laplace approximations (INLA) with the R INLA package (Rue et al. 2013; Blangiardo et al. 2013). The study protocol was approved as exempt by the New York University School of Medicine Institutional Review Board.

## Results

**Fig. 1:**
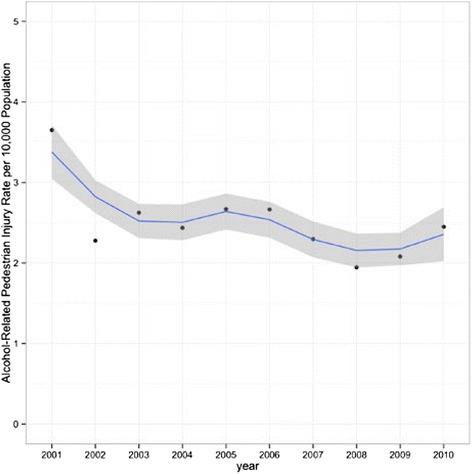
Yearly Alcohol-Related Pedestrian and Bicyclist Injury Rate per 100,000 Population With Overlying Loess Smoothing Line. New York City, 2001–2010

Of the 168,060 pedestrian and bicyclist injury records for New York City between 2001 and 2010, 93,783 (55.8 %) had a valid entry for a primary apparent contributing factor. 2,170 of these records listed alcohol as the primary apparent contributing factor. Of the 1,908 New York City census tracts included in the study 198 (10.4 %) had at least one actively licensed alcohol outlet. The population-based alcohol-related pedestrian/bicyclist injury rate varied by borough. The highest rate was in Manhattan was 33.9 injuries per 100,000 population, followed by Brooklyn with 27.6 injuries per 100,000 population. The boroughs of Queens and the Bronx had similar rates, with 14.6 and 14.9 injuries per 100,000 population respectively. The lowest rate was in Staten Island, with 7.0 injuries per 100,000 population. There was an initial drop in the population-based rate of alcohol-related pedestrian injuries in New York City in 2002, followed by a relatively stable rate of approximately 2.8 alcohol-related injuries per 100,000 persons each year (Fig. 1).

The results for the fixed effects of the presence of alcohol outlets and covariates for the the spatial model are presented in Table 1. Holding all other covariates to zero and adjusting for both random and spatial variation, the presence of a at least one alcohol outlet in a census tract increased the risk of a pedestrian or bicyclist being struck by a car by 47 % (IDR = 1.47, 95 % Credible Interval (CrI) 1.13, 1.91). Every one unit increase in the social fragmentation index was associated with a 25 % increase in pedestrian injury risk (95 % CrI 1.17, 1.35). Similarly, for every one standardized unit increase in traffic density, there was a 28 % increase in pedestrian injury risk (95 % CrI 1.16, 1.40). For every 10 mile per hour increase in average traffic speed in a census tract, there was a 31 % decrease in pedestrian injury risk (95 % CrI 0.55, 0.87). Each $10,000 increment in median household income within a census tract was associated with a 1 % decrease in census-tract level pedestrian injury risk (95 % CrI 0.98, 0.99).

**Table 1:** Incidence Density Ratios (IDR) for risk of pedestrian bicyclist injury in relation to built environment characteristics, New York City, 2001–2010

	IDR	95 % credible interval
At least one alcohol outlet	1.47	(1.13, 1.91)
Social fragmentation	1.25	(1.17, 1.35)
Median household income (per $10,000)	0.99	(0.98, 0.99)
Traffic density, z-score	1.28	(1.16, 1.40)
Average vehicle speed, per 10 MPH increment	0.69	(0.55, 0.87)

**Fig. 2:**
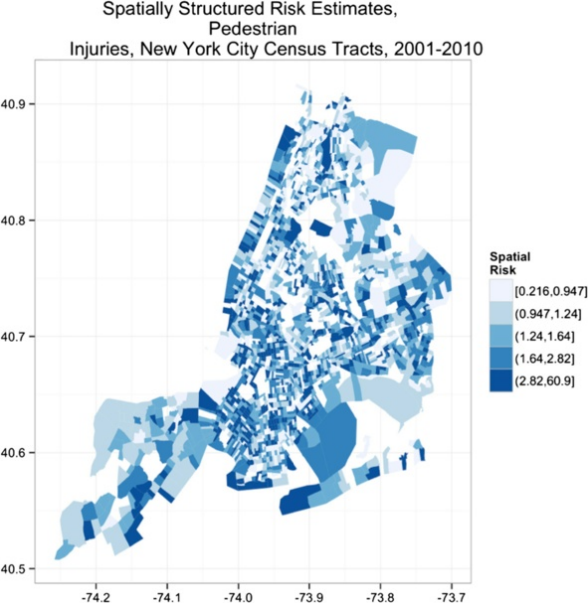
Relative risk of alcohol-related pedestrian injury, New York City census tracts 2001–2010

**Fig. 3:**
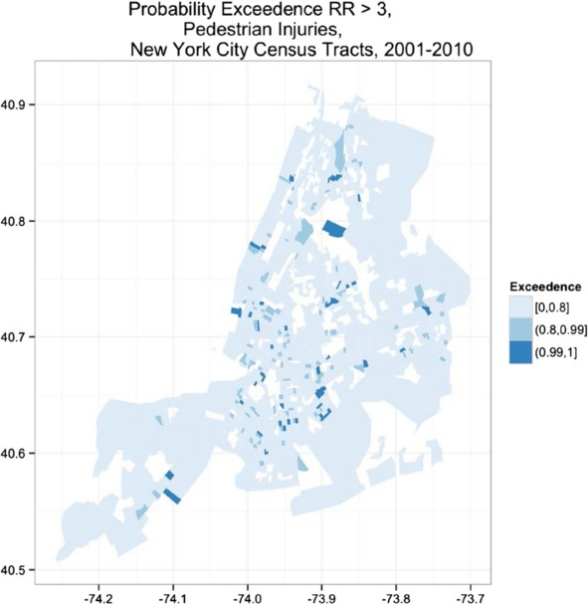
Probability of relative risk of alcohol-related pedestrian or bicyclist motor vehicle injury greater than 3, New York City census tracts 2001–2010

**Fig. 4:**
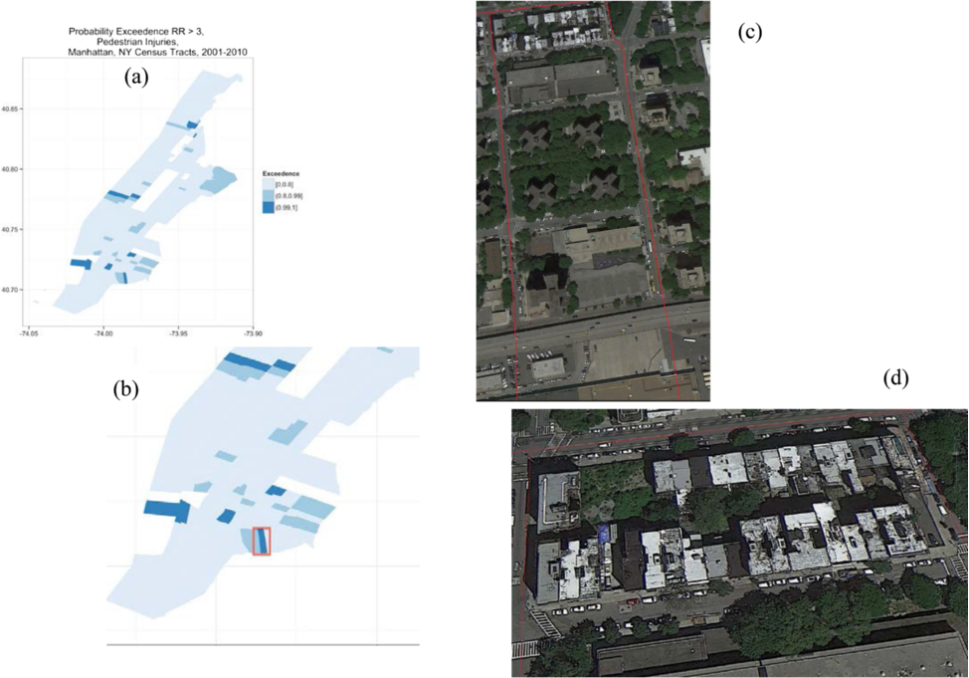
**a** Probability map of alcohol-related pedestrian or bicyclist injury relative risk greater than 3 in Manhattan, **b** Single census tract with at least 85 % probability of relative risk greater than 3, **c** Zooming in on the census tract, **d** General built environmental characteristics of census tract. Alcohol-related pedestrian and bicyclist injuries, New York City census tracts, 2001–2010

**Fig. 5:**
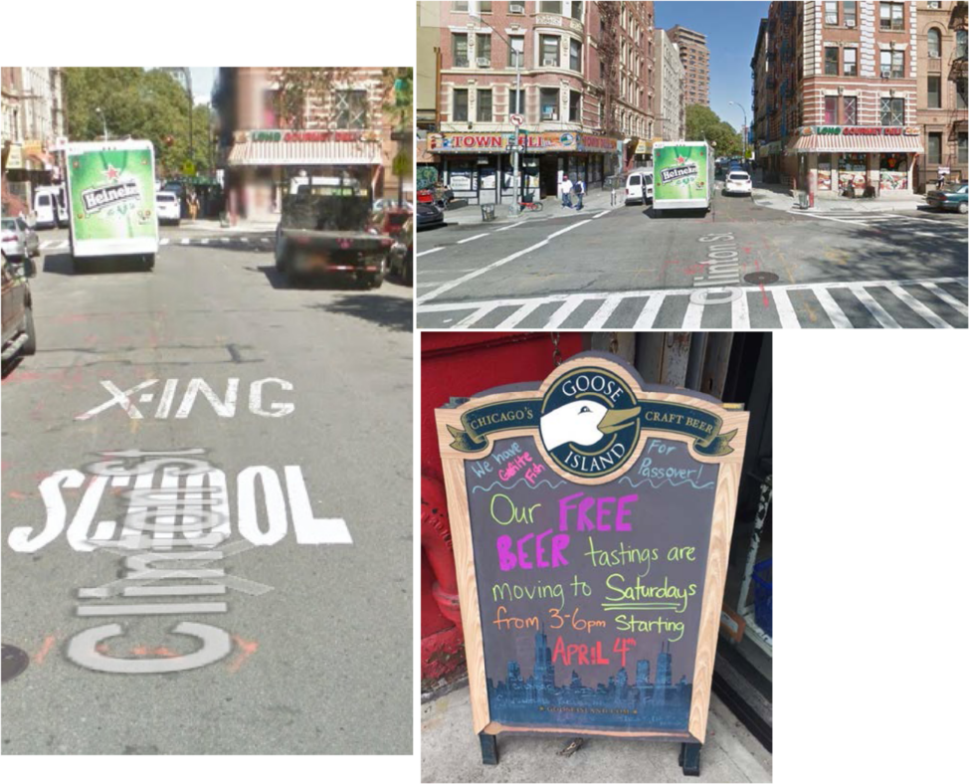
Google Street View images from selected Manhattan census tract with at least 85 % probability of relative risk of alcohol-related pedestrian or bicyclist injury greater than 3

The spatially unstructured heterogeneity random effect term was normally distributed and spatially random. The spatially structured conditional autoregression term demonstrated areas of spatial patterning and similarity among census tracts. The proportion of the total spatial heterogeneity explained by the spatially structured conditional autoregression term was 73.2 %. The spatial risk term at the census tract level identified areas at increased risk of alcohol-related pedestrian/bicyclist injury throughout the 10-year period (Fig. 2). Posterior probabilities for the 48 census tracts with a spatial risk estimate of alcohol-related injury exceeding 3 are presented in Fig. 3. In this figure, for each census tract highlighted in dark blue, the probability that the risk that a pedestrian or bicyclist injury is alcohol-associated is greater than 3 approaches near certainty. In Fig. 4, a single census tract from lower Manhattan is selected where the probability is greater than 85 % that the risk of an injury is alcohol-related injury is at least 3. A satellite image for this tract obtained from Google Maps is then presented, illustrating the general physical layout of the tract. Figure 5 then presents the a detailed Google Street View image of one of the blocks in the census tract, illustrating the kind of detail available to researchers and persons conducting public health evaluations of the built environment for features related to pedestrian injury.

## Discussion and conclusions

In this study, we found that presence of alcohol outlets was meaningfully associated with the risk of alcohol-related pedestrian and bicyclist injury in New York City from 2001 to 2010. Alcohol consumption, whether on the part of a motor vehicle driver or a vulnerable road user, increases the likelihood of a crash, the severity of injuries incurred in a crash, and the odds of death due to a crash (Peden and Sminkey 2004).

Drinking places not only the driver and vehicle passengers at risk for serious bodily harm but also pedestrians and cyclists who share the road. Although vehicle safety upgrades (e.g. air bags, structural integrity) and educational and legislative campaigns (e.g. seatbelt use, child seats) have made driving safer and reduced the number of deaths of motor vehicle occupants, studies from both the US (MMWR 2001) and Australia (Holubowycz 1995) indicate that alcohol-related pedestrian collisions and deaths have not decreased to the same extent. Injured pedestrians who have used alcohol suffer more severe injury, with statistically significantly higher injury severity scores (8.8 vs 4.9) and length of stays (3.9 vs. 1.9 days) (Dultz et al. 2011). In 2009 in the United States, 53 % of pedestrians killed between 9 PM and 6 AM had blood alcohol concentrations (BACs) at or above 0.08 % (NHTSA 2010).

Our study was motivated in part by the recommendation that “The main elements of (a strategic) approach involved systematic methods for identifying locations where the problem is greatest and/or more acute…” (Corben et al. 1996). While our analyses replicate others that have shown that areas around bars or other establishments which serve alcohol are frequent hotspots for pedestrian injury (Schuurman et al. 2009), they further quantify that risk, place it in the commonly utilized spatial perspective of a census tract, and demonstrate a methodological approach to surveillance of alcohol-related pedestrian and bicyclist injuries in urban environments.

We believe this work adds to the existing research on pedestrian injury epidemiology in three ways. First, the study presents alcohol-related pedestrian and bicyclist injury risk assessment at a finer level of geographic detail than has been generally been reported, which allows more opportunity for focused investigations and interventions. Second, the methods in this study, which have previously not been used for alcohol-related pedestrian injury research, offer practical tools for spatial analysis in injury epidemiology that might not otherwise be readily apparent. Finally, the study extends the well-recognized association of pedestrian injury with alcohol intoxication by quantifying pedestrian and bicylist injury’s association with alcohol outlets (Öström and Eriksson 2001). Furthermore, the socioeconomic and traffic-related control variables may themselves offer additional insights into the complex interplay of physical and sociocultural environment in pedestrian injury risk.

We found place, in particular the presence of alcohol outlets in a census tract, to be a critically important determinant of alcohol-related pedestrian and bicyclist injury risk beyond other perhaps, more immediately apparent factors such as traffic density. There were areas in each borough of New York City where the risk of alcohol-related pedestrian injury was higher than the city as whole throughout the 10-year study period. The use of exceedance probabilities refines this characterization of risk by placing explicit probabilities on the observed risk estimates to identify those areas for which the increased risk was highly unlikely to be due to chance.

Injury risk at the census tract level was associated with social, economic and traffic-related factors. Census tracts benefited unequally from the over-all reductions, with some areas having consistently elevated risks compared to the city-wide experience, and others experiencing sporadic years of increased risk. The kinds of small-area spatiotemporal Bayesian hierarchical modeling approaches used in this study are increasingly a practical option for epidemiologists interested in evaluating risk of disease outcomes in the context of place and time.

Pedestrian injury risk decreased with increasing average vehicle speed in a census tract. There are a number of possible explanations for this. We do not know the speed of the vehicles that struck pedestrians, so while the average speed was lower, the vehicles striking pedestrians may have been traveling at higher than average speeds. In addition, slower speeds may indicate more vehicles, which increases overall exposure to traffic. It may also be that because the average vehicle speed and traffic density variables in these analyses included highways, bridges and tunnels, areas with higher average speeds would be less likely to be frequented by pedestrians, decreasing exposure. A sensitivity analysis using a version of the speed and density variables that did not include highways, bridges and tunnels, though, returned essentially the same results. This may also be an artifact of measurement error due to using census tract population as a proxy for pedestrian population. Some tourist and retail-oriented neighborhoods, such as Greenwich Village and SoHo, have very high pedestrian counts and very low vehicle speeds. Finally, average speeds are a group-level measure aggregated from individual measurements; though analysis of measures aggregated from continuous variabels are less prone to bias away from the null than aggregate from dichotomous measures, small amounts of non-differential measurement error at the individual level may lead to bias away from the null at the group level if fewer measurements contribute to the aggregate measure (Mooney et al. 2014).

We believe our model represents an acceptable trade off between goodness of fit and complexity, and that the variables we included capture much of the potential confounding effects associated with areas where alcohol establishments might be typically located. As a sensitivity analysis for this, we re-ran the model including a variable for the proportion of the total area of space in a census tract proportion of space in a census tract accounted for by retail activity (NYC Department of City Planning 2016; Neckerman et al. 2009). The addition of this variable did not change our original results in any meaningful way. We ran a further analysis with a measure of the health characteristics of the local retail food environment in a census tract obtained from the Centers for Disease Control and Prevention (CDC 2011). The results were similarly unchanged.

Ideally, external validation would include the onsite evaluation of census tracts determined by initial analyses to have a high probability of being at higher-than-average risk of pedestrian injury. Site visits can yield deep and practical insights about local risks (Hameed et al. 2004), but are expensive, time consuming and require complex sampling strategies. Virtual site visits using tools like Google street view to collect and evaluate local geographic data with less expense and difficulty have been proposed and demonstrated. While we attempted to illustrate the results using satellite images of a single census tract in our study, formal validation efforts would include statistical approaches, such as comparing predictions based on the model to actual data from years beyond 2010, or could involve statistical associations with external sources of covariate data. In one evaluation of fixed local covariates, the increased frequency of pedestrian crashes in low income, high minority areas in Chicago was shown to be associated with walkability and access to transit options (Cottrill and Thakuriah 2010).

There are a number of important limitations in this study. We could not determine whether alcohol contributed to the risk of injury through the pedestrian/bicyclist or the driver. Alcohol outlets were tightly clustered in our sample, with 22,464 licenses located within 198 census tracts. For this reason, we chose to use a binary indicator for the presence or absence of a licensed outlet in a census tract, rather than the incremental effect of each additional licensee. We restricted our analyses to only those incidents in which law enforcement clearly determined alcohol to have been a primary contributing factor. We felt this strengthened the specificity of any result, but it is likely that additional alcohol-related injuries occurred. Having 2000–2010 crash risks predicted by 2015 alcohol outlets and 2010 income is not optimal. There is some evidence that the number of alcohol outlets in New York City have been increasing, so this will likely bias these effects toward zero (Fickenscher 2014). Future research on the spatial distribution of alcohol-related pedestrian injury might benefit from use of longitudinal business registration data, such as that offered by the National Establishment Time-Series dataset (Walls 2011).

While the Bayesian CAR model we used “draws strength” from surrounding areas and is a way to smooth these edge effects, future analyses could profitably include opportunities to look at spatially-lagged impacts on census tracts that perhaps do not have alcohol outlets but are adjacent to those that do have alcohol outlets. These kind of effects may evidence as patterns in drinking and driving arrests. Additionally, we did not, in this analysis, consider the different types of alcohol outlets in a census tract. We believe this first analysis must necessarily address the universe of alcohol outlets before parsing out effects based on subpopulations. We similarly plan to conduct such analyses in the future.

Two of the statistical approaches used in this study also come with caveats. First, exceedance probabilities, which have been proposed as a Bayesian approach to hotspot identification and are in relatively common use, (Hossain and Lawson 2006; Hossain and Lawson 2010) can be sensitive to model specifications (Lawson 2006). And second, the proportion of variance explained by the spatially structured conditional autoregression term is not strictly speaking a variance partition coefficient, because the structured and unstructured spatial terms may not be directly comparable. It is, though, an indication of the relative contribution of each of the spatial components.

There were 2,217 census tract in 2000, and 2,168 tracts in 2010, for an overall decrease of 2.2 %. There were 288 census tracts in the 2000 data set that are not in the 2010 data set. There were conversely 239 census tracts in the 2010 data set, that were not in the 2000 data set. To ensure the consistency and reliability of the population data across the 10 years of study, and across the analytic approaches that took geography into account, we restricted the analyses to those census tracts that were present in both census years. This resulted in some raggedness to the adjacency matrix, but we felt allowed for more valid comparisons across space and time, and between intervention and non-intervention sites. Finally, the boundary line between census tracts is typically a street; in our study, any collision that fell on such a street has been assigned to one tract or the other based on the coordinates reported in records. We expect that any error due to boundary effects is absorbed into the spatial autoregressive error term and results in a bias towards the null.

We conclude that the presence of one or more alcohol outlets in a census tract in an urban environment increases the risk of bicyclist/pedestrian injury in important and meaningful ways. This increased risk is beyond what might be expected based on associated variables like vehicle and pedestrian density, or socioeconomics. Identifying areas of increased risk due to alcohol allow the targeting of interventions to prevent and control alcohol-related pedestrian and bicyclist injuries.
